# Assessment of adulthood immunization knowledge, attitudes, and behavior

**DOI:** 10.1590/1806-9282.20241779

**Published:** 2025-05-02

**Authors:** Özgür Özerdoğan, Sibel Oymak, Coşkun Bakar

**Affiliations:** 1 Canakkale University, Faculty of Medicine, Department of Public Health – Çanakkale, Turkey.

**Keywords:** Adult, Vaccine, Immunization

## Abstract

**OBJECTIVE:**

Adulthood vaccination has not reached adequate levels, both in Turkey and around the world. The aim of this study was to identify the knowledge, attitude, and behavior of vaccination in those aged 18 years.

**METHODS:**

This is a cross-sectional study. Questionnaires were applied to 686 participants attending Family Health Centers. For the analysis of data, the statistical significance used was p<0.05.

**RESULTS:**

Notably, 72.4% of people had at least one vaccination in adulthood. The most frequent vaccinations were tetanus (55.1%), influenza (26.8%), and hepatitis B (8.2%). PATH analysis found that the effect of variables with direct effects on vaccination (apart from the situation of thinking that vaccinations are necessary in adulthood) disappeared in the model in which adult vaccine recommendations were used as mediators.

**CONCLUSION:**

The adult vaccination situation is inadequate. It is necessary to inform society about adult vaccinations and recommend vaccination. Tools such as information given during health services and implementations such as social education and brochures, posters, media, and public information spots may be used with this aim.

## INTRODUCTION

Immunization acquired by vaccination is one of the most effective strategies to protect public health. Within the framework of the Expanded Programme on Immunization recommended by the World Health Organization, significant progress has been made for vaccinations in the newborn and childhood periods on a global level. But it is not possible to say the success of neonatal and childhood vaccinations in the world in general is reflected in vaccination in the adult^1-3^. Whereas, especially due to reasons such as pregnancy, chronic diseases, travel, and intravenous drug use, susceptible groups emerge and require vaccination in adulthood^4^. Mortality and morbidity due to diseases preventable with vaccinations in the adult are still observed. For example, every year nearly 50,000 adults die due to diseases preventable with vaccination in America^5^.

In Turkey, there is no systematic program about vaccination in the adult period, apart from some vaccinations administered to women of reproductive age and the elderly. Studies have shown that the public does not have sufficient information about this topic^6^. This research was completed with the aim of investigating the knowledge, attitude, and behavior of adult individuals about the topic.

## METHODS

This study was a cross-sectional study. The research was completed in Canakkale province located in the Southern Marmara region of Turkey.

The population comprised individuals aged 18 years and older residing in neighborhoods in Kepez town located in Canakkale central county and linked to the central county. The sample size formula was used to estimate the sample size population rate. This formula used a value of p: 0.05 in situations where the rate is not known for the incidence of an event (Zα/2:1.96, alpha value 0.05, deviation 5%). The population value was taken as the population aged 18 years and older residing in neighborhoods in Canakkale central county and Kepez town (126,893), and the minimal sample number to be reached was determined as 384 people. The minimal sample number that must be reached was weighted for the population aged 18 years and older in neighborhoods in the research region with the layered sample selection method. Notably, six family health centers (FHC) representing all neighborhoods in the research region were chosen. People attending the FHC were reached on a voluntary basis with the non-probability sampling method. The research was completed in six FHC in August 2019. In the period of the study, 686 people from a total of 1,311 aged 18 years and older attending the FHC (52.3%) were reached (more people were reached in each neighborhood than the minimal sample number). With the face-to-face interview method, participants completed a survey lasting 10–15 min. The survey included questions about sociodemographic features, questions about current health status and information, and attitudes and behavior about adult immunization.

### Ethics committee approval

Written permission for the study was granted by Canakkale Provincial Directorate of Health for the FHC in the study (number: 18231034-604.99) and by Canakkale Onsekiz Mart University clinical research ethics committee (decision date: 20.02.2019, decision no: 2019-04).

### Statistical analysis

Analysis of research data used the SPSS version 20.0, STATA 14, and SPSS AMOS programs. Analyses accepted p<0.05 as statistically significant.

PATH analysis (adulthood vaccination recommendations as a mediator variable) was applied to investigate factors that may affect the situation of having any vaccination in adulthood. PATH analysis is a form of multiple regression describing the indirect, direct, and total effects of independent variables on a dependent variable. To assess the fit of the model, acceptable limits were 0.05<RMSEA£0.08, 0.05<RMR£0.08, 0.90£FI£0.94, 0.90£CFI£0.94, 2<X^2^/sd£5, while limits of perfect fit were 0£RMSEA£ 0.05, 0£RMR£0.05, GFI≥0.90, AGFI≥0.90, NFI≥0.95, IFI≥0.95, CFI≥0.95, 0£X^2^/sd£2. The applied PATH analysis model abided by these criteria.

## RESULTS

The study group comprised 686 people. Mean age was 48.1±16.5 years (median: 49, minimum–maximum: 18–90). Other descriptive features of participants are presented in Table 1.

**Table 1:** Characteristics of the study group.

Variables		Mean (median)
Age (year) (n: 686)		48.1±16.5 (49.0)
Gender (n: 686)		**n (%)**
	Female	358 (52.2)
	Male	328 (47.8)
Occupation (n: 686)		
	Healthcare professionals/healthcare workers	41 (6.0)
	Other occupational groups	645 (94.0)
Marital status (n: 686)		
	Married	505 (73.6)
	Single	119 (17.3)
	Widow/divorced	62 (9.1)
Education status (n: 686)		
	Primary education	271 (39.5)
	High school and higher education	415 (60.5)
Chronic illness (n: 686)		
	Yes	376 (54.8)
	No	310 (45.2)
Allergy to any factor (n: 686)		
	Yes	152 (22.2)
	No	534 (77.8)

n: number; %: percentage.

Of the study group, 77% saw vaccination in the adult as required and 72.4% had done any vaccination in the adult (Table 2). Of vaccinations done, the most common were tetanus (55.1%), influenza (26.8%), and hepatitis B (8.2%).

**Table 2:** Vaccination in adulthood, recommendation to vaccination, opinions about vaccination, and childhood vaccination status.

Variables		n (%)
Thinking that vaccination is necessary in adulthood (n: 686)		
	Yes	528 (77.0)
	No	80 (11.7)
	Undecided	78 (11.3)
To be vaccinated in adulthood (n: 686)		
	Yes	497 (72.4)
	No	189 (27.6)
Recommendation for vaccination in adulthood (n: 686)		
	Yes	404 (58.9)
	No	282 (41.1)
Being of proposed adulthood vaccination (n: 404)*		
	Yes	366 (90.6)
	No	38 (9.4)
Ideas on adulthood vaccination (n: 686)**		
	I know about adulthood vaccinations; however, I don't know what these vaccinations are	351 (51.2)
	Only adults with some diseases should be vaccinated, there is no need to vaccinate all adults	263 (38.3)
	I know exactly of adulthood vaccinations	41 (6.0)
	There isn't need for vaccination in adulthood	24 (3.5)
	Vaccinations are required for only children	19 (2.8)
	All adults should be vaccinated	27 (3.9)
	Required only in case of epidemic	3 (0.4)
	I have no idea	30 (4.4)

n: number; %: percentage;

* Participants who were recommended to be vaccinated in adulthood answered this question;

** More than one answer could be obtained for this question and the percentage was calculated from the study group of 686 people.

Among participants, 58.9% were recommended vaccinations in the adult and 9.4% of these people had not done the recommended vaccines (Table 2). When the reasons for rejection were asked of these 38 people who had not done the recommended vaccines, the most common responses were not thinking the vaccines were protective against disease (26.3%), not thinking the vaccination was necessary/thinking vaccines are harmful (23.7%), thinking that they would acquire immunity by catching the disease rather than from vaccination (21.1%), fear of vaccine side effects (10.5%), and not having time to get the vaccine (10.5%).

When people were asked about their thoughts on adulthood vaccinations, the most frequent responses were, "I know about adulthood vaccinations; however, I don't know what these vaccinations are" (51.2%) and "only adults with some diseases should be vaccinated, there is no need to vaccinate all adults" (38.3%). Among participants, 63.3% had received vaccines in the childhood period and 73.2% stated that they or their family did not have their childhood vaccine card (Table 2). When people were asked, "whose opinion and recommendation is important to you in your decision to be vaccines?," the most common answer was health employees at 92%.

Factors that may affect vaccination in the adult were investigated with PATH analysis. Variables explaining direct effects were reduced age, being male, being married, having chronic disease, having allergies, thinking vaccination in adults will protect infants, children, and pregnant people around them from disease, they or their families not having their childhood vaccine card, seeing vaccinations as necessary in the adult, and receiving vaccinations recommended to them in the adult (p<0.05). In the model where being recommended vaccination in the adult was used as the mediator variable (indirect effects), explanatory variables were identified as being a health employee/working in the health area and seeing vaccination as necessary in the adult (p<0.05) (Table 3).

**Table 3:** Affecting factors of vaccination in adulthood, PATH analysis.

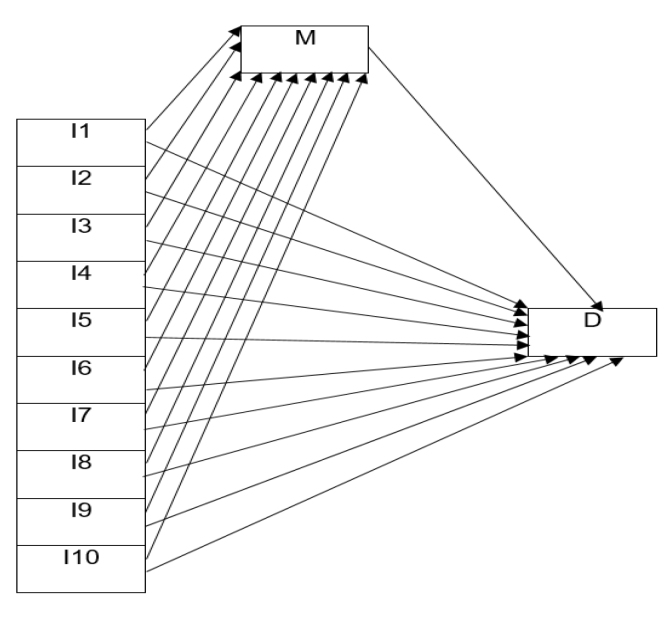	**Direct effect**		**EC**	**p-value**	**Indirect effect**		**EC**	**p-value**
		M->D	0.5231	**<0.001**				
		I1->D	-0.0031	**0.005**		I1->M->D	-0.0014	0.085
		I2->D	0.0704	**0.010**		I2->M->D	-0.0071	0.719
		I3->D	0.0618	0.275		I3->M->D	0.0894	**0.030**
		I4->D	0.0458	**0.014**		I4->M->D	0.0116	0.394
		I5->D	0.0648	**0.043**		I5->M->D	-0.0072	0.755
		I6->D	0.1088	**0.001**		I6->M->D	-0.0011	0.961
		I7->D	0.0562	**0.003**		I7->M->D	0.0194	0.165
		I8->D	0.0647	0.050		I8->M->D	-0.0449	0.061
		I9->D	0.0552	**0.008**		I9->M->D	0.0005	0.974
		I10->D	0.0491	**0.014**		I10->M>D	0.0564	**<0.001**

I: Independent variable; I1: Age (continuous variable); I2: Gender (female: 0, male: 1); I3: Occupation (other occupational groups: 0, healthcare professionals/healthcare workers: 1); I4: Marital status (single: 0, widow/divorced: 1, married: 2); I5: Chronic illness (no: 0, yes: 1); I6: Allergy (no: 0, yes: 1); I7: Thinking that vaccination in adults will protect infants, children, and pregnant people around them from disease (undecided: 0, no: 1, yes: 2); I8: Childhood immunization status (All childhood vaccinations have been/have been done, but doesn't know whether all of them have been done: 0, Doesn't know/remember any one, no: 2); I9: Having the their childhood vaccination card in themselves or their family (yes: 0, doesn't know/remember: 1, no: 2); I10: Thinking that vaccinations are necessary in the adulthood (no: 0, undecided: 1, yes: 2); M (Mediator variable): Recommendation to vaccination in adulthood (no: 0, yes: 1); D (Dependent variable): To be vaccinated in adulthood (no: 0, yes: 1); EC: effect coefficient; p: statistical significance level.

In statistical analysis, p<0.05 is statistically significant. p<0.05 values are bold.

## DISCUSSION

The majority of participants (77%) saw vaccination in the adult as necessary. A study performed in FHC in Ankara found this frequency was 50.5%, while a study in a family clinician clinic in an education-research hospital identified this rate was 84.3%^7,8^. Different results obtained about this topic lead to consideration that there are attitude variations in geographies with different sociocultural features. The incidence of receiving any vaccine in the adult is 72.4%. The most common vaccinations were tetanus (55.1%), influenza (26.8%), and hepatitis B (8.2%). A study in the Istanbul region reported that 57.9% had done vaccinations in the adult and the most common vaccinations were tetanus (42.1%), influenza (23.9%), and hepatitis B (18.2%)^9^. A study by Bolatkale et al. identified that the most common vaccinations were tetanus (59%), influenza (35.1%), and hepatitis B (28.1%)^8^. In Turkey, tetanus vaccinations are administered in emergency services as a result of injury, within the framework of the maternal and neonatal tetanus elimination program and during military duty. The high identification of the influenza vaccine administration frequency may be explained by the occasional deadly influenza epidemics observed and the awareness of this situation in society. However, the vaccination frequencies obtained in studies are not at the desired levels.

In this study, 58.9% of people had been recommended vaccinations in adulthood. Of those recommended vaccinations, 9.4% had not done vaccines for a variety of reasons. The most common reasons were not thinking vaccines were protective against the disease; not seeing vaccination as required/thinking vaccines are harmful; thinking they would acquire immunity by catching the disease rather than from vaccination; and fear of vaccine side effects. A study by Uzuner et al. found the most common responses to the question "if you did not have the vaccinations, what was the reason?" were not being informed about the topic (47.1%), not seeing vaccination as required (43.2%), and thinking of vaccine side effects (3.1%)^9^. Non-scientific misinformation has an important place in vaccine rejection. Positive policies should be developed to inform the public.

Half of our study group had information about adulthood vaccinations; however, they did not know what these vaccinations were, with 38.3% stating vaccinations were only required for those with some illnesses, 2.8% thinking vaccinations were only required by children, and 4.4% having no idea about the topic. A study in Antalya stated that 37% of people thought only those with certain diseases required vaccination, 36% knew about adulthood immunization but did not fully know what, 4% thought vaccinations could only be administered to children, and another 4% stated that vaccination was not required in the adult^10^. Studies show that society does not have sufficient information about adult vaccines, and there is common misinformation that vaccines are only required for some diseases. In our study, when people were asked, "whose opinion and recommendations are most important to you when deciding on vaccination?" the most common response was health employees (92%). In the study by Karabay et al., 93.8% of the participants answered that the guidance of healthcare professionals was effective in getting vaccinated^6^. A study conducted in Ankara revealed that when participants were asked about their preferred sources for informed on adulthood vaccinations, the most common responses were doctors (88.0%) and midwives/nurses (25.4%)^7^. The frequent misinformation in society and seeing health employees as a reliable source about the topic indicate just how important it is for health employees to give information about adulthood vaccination in every situation of contact with patients. Another study found that only 20.5% of clinicians had done information about adulthood vaccination after graduation and the majority (88.5%) stated they required practical refreshment^11^. We believe that education for health personnel before and after graduation will be effective in communicating accurate information to society. Informing health employees, the most important source of information in society, with frequent in-service education and about making vaccination recommendations to patients may contribute to developing positive attitudes. Social media may be used with this aim. However, it is important for society to reach information about evidence-based medical practices through the use of social media. Otherwise, there may be information pollution about the topic on social media. For this reason, we think it is important that scientific organizations or scientists use social media more frequently with the aim of transferring information to society.

In the literature, the factors affecting vaccination in adults display variability. For example, the study by Asık et al. identified that being a woman was a factor increasing vaccination, while age was ineffective^10^. Similarly, a study in Ankara stated that the gender and age variables did not affect vaccination in the adult^7^. In our study, factors affecting vaccination in the adult were investigated with PATH analysis (direct and indirect effect). Indirect effects appeared to remove the effects of a variety of sociodemographic factors (like age, gender, and presence of chronic disease) as a result of vaccine recommendations to people. This situation may indicate that people tend to get vaccinated when recommended, regardless of sociodemographic factors. When examined from this aspect, one of the most important variables affecting vaccination behavior is recommending vaccines to people. In studies in the literature, one of the most important reasons for the lack of sufficient levels of adulthood vaccination is the lack of vaccine recommendations^12^. A study conducted at Akdeniz University Faculty of Medicine Hospital involving over 2,000 individuals found that 68.2% of those vaccinated received their vaccines based on recommendations from their doctor^13^. Additionally, a separate study at Yozgat Bozok University, which included 3,000 participants, revealed that 73.7% of individuals who were recommended pneumococcal vaccinations and 68.4% of those who were recommended influenza vaccinations went on to receive those vaccinations^14^. People can be offered vaccines through healthcare professionals. Social media, television, public information spots, and public education studies may be used to recommend vaccinations to the public.

## CONCLUSION

The frequency of vaccination in adults, as well as their awareness and attitudes toward this issue, is currently insufficient. One of the key factors that can increase vaccination rates is the recommendation of vaccines for individuals. To address this, it is essential to continuously provide information during health service implementations and public education initiatives. Additionally, enhancing the importance of vaccination in the training of healthcare personnel is crucial. Tools such as brochures, posters, and public information campaigns can be effectively utilized for this purpose.
